# Breaking Barriers in Stroke Therapy: Recent Advances and Ongoing Challenges

**DOI:** 10.7759/cureus.78288

**Published:** 2025-01-31

**Authors:** Akkila Goud Bakka, Shweta Shirish Patil, Bhavagna Rachakonda, Abhishek Patil, Jahnavi Bolleddula, Prasanna Sai Kumar Reddy Nandyala, Reva Singamaneni, Nagasaikaran Sade, Prashanth Kumar Patnaik

**Affiliations:** 1 Department of Pharmacology, RVM Institute of Medical Sciences and Research Center, Laxmakkapally, IND; 2 Department of Internal Medicine, Shri Bhausaheb Hire Government Medical College, Dhule, IND; 3 Department of Medicine, RVM Institute of Medical Sciences and Research Center, Laxmakkapally, IND; 4 Department of Internal Medicine, Apollo Institute of Medical Sciences and Research, Hyderabad, IND

**Keywords:** biomarkers, ischemic stroke, mechanical thrombectomy, stroke management, thrombolytic therapy

## Abstract

Acute ischemic stroke remains a significant global health challenge, prompting notable advancements in its management. Diagnostic innovations, including advanced imaging techniques and biomarker discovery, have greatly improved early detection and treatment precision. Mobile stroke units have expedited pre-hospital care, emphasizing the urgency of intervention under the principle "time is brain." These developments underscore the importance of timely and accurate diagnosis in improving outcomes.

Therapeutic breakthroughs in thrombolytic therapy and mechanical thrombectomy have transformed acute stroke treatment. Refined thrombolytic protocols, new agents, and extended treatment windows have expanded therapeutic possibilities. Mechanical thrombectomy, bolstered by improved devices and techniques, has achieved higher recanalization rates and broader applicability, solidifying its role in endovascular interventions. Meanwhile, post-stroke care has embraced personalized, multidisciplinary rehabilitation approaches, with telemedicine breaking geographical barriers and enhancing recovery through patient-centered models.

Despite these advancements, challenges persist, including disparities in access to advanced care and the underexplored management of minor strokes. Emerging technologies, such as artificial intelligence and machine learning, offer opportunities for better risk prediction and earlier interventions, though their long-term impacts require further study. This review highlights the transformative progress in stroke care while emphasizing the need for continued innovation, research, and equitable access to ensure better outcomes for all patients.

## Introduction and background

Stroke remains a significant global health challenge, with ischemic stroke being the most common type of cerebrovascular disorder. It is primarily caused by arterial blockage, leading to significant neurological impairment and imposing a substantial burden on patients, families, and healthcare systems worldwide [1,2]. The global impact of stroke transcends geographic and socioeconomic boundaries, affecting millions annually and straining healthcare resources. Ischemic stroke accounts for the majority of cases, highlighting the urgency for effective prevention and management strategies [3]. Early detection and timely intervention remain critical for reducing mortality and improving recovery outcomes. Recent years have seen significant progress in diagnostic tools, therapeutic approaches, and rehabilitation strategies, reshaping the outlook for stroke patients [4-6].

Advances in diagnostic imaging, such as high-resolution magnetic resonance imaging (MRI) and computed tomography (CT) technologies, have improved the accuracy and speed of identifying ischemic strokes, enabling earlier intervention [7]. Novel biomarkers are enhancing the detection of at-risk patients and helping clinicians tailor treatments more effectively [8,9]. Mobile stroke units (MSUs) and telemedicine have emerged as transformative innovations in stroke care, significantly improving access to timely and specialized treatment, particularly in underserved and remote regions. MSUs are specialized ambulances equipped with advanced imaging technologies, such as portable CT scanners, and staffed by trained healthcare professionals, including neurologists and paramedics. These units enable rapid on-site diagnosis and the initiation of treatment, such as administering clot-busting drugs, long before the patient reaches the hospital. Similarly, telemedicine facilitates real-time consultation with stroke specialists through videoconferencing, allowing immediate guidance for diagnosis and treatment decisions in areas lacking access to neurologists [10]. These advancements embody the principle of "time is brain," a concept emphasizing that every moment counts during a stroke. In ischemic strokes, the brain is deprived of oxygen and nutrients due to a blocked artery, and as a result, approximately 1.9 million neurons are lost every minute without treatment. This rapid neuronal loss underscores the critical importance of minimizing delays in diagnosis and intervention to preserve brain function and improve patient outcomes. MSUs and telemedicine directly address these challenges by reducing pre-hospital delays, ensuring the faster delivery of evidence-based treatments, and improving survival rates and long-term recovery prospects [10,11].

Therapeutic advancements, including refinements in thrombolytic therapy and the expanding use of mechanical thrombectomy, have transformed acute stroke treatment. Extended treatment windows and improved techniques have increased the number of patients eligible for these life-saving interventions [11]. Furthermore, neuroprotective agents and anti-inflammatory therapies are under investigation, offering potential new avenues for treatment [11].

Post-stroke care has embraced personalized rehabilitation strategies, integrating multidisciplinary teams to address physical, cognitive, and emotional recovery. Tele-rehabilitation has emerged as a crucial tool, overcoming geographic barriers and providing continued care for stroke survivors. Patient-centric approaches now emphasize shared decision-making and caregiver involvement, improving overall outcomes and quality of life [9-11]. Emerging technologies such as artificial intelligence (AI) and machine learning hold promise for improving early risk prediction and intervention but require further validation in clinical settings [9-11]. The management of minor strokes and transient ischemic attacks also demands more attention, as these often signal larger vascular risks [11].

In summary, acute ischemic stroke management has entered a transformative era, driven by collaborative efforts across research, technology, and clinical practice. This review highlights the strides made while acknowledging the need for equitable access, ongoing innovation, and a patient-centered approach to care. The journey of stroke management continues, with promising developments on the horizon and a steadfast commitment to improving outcomes for those affected by this life-altering condition.

## Review

Data sources

This investigation employed a meticulous and comprehensive search across prestigious medical databases, recognized for their pivotal contributions to medical science. The primary sources included PubMed, Embase, and the Cochrane Library, selected for their reputation as repositories of rigorously peer-reviewed research and their extensive curation of scholarly work in medicine. The temporal span of this review was intentionally set to cover a decade, from 2013 to 2023. This timeframe was chosen to provide a broad yet focused examination of contemporary progress, encompassing both recent advancements and the latest contributions in the field of acute ischemic stroke management.

Search strategy

The search strategy was carefully formulated to retrieve a wide range of studies, covering various aspects of acute ischemic stroke management. The key terms included the following: "Acute Ischemic Stroke", the primary focus of our study which is crucial for identifying relevant research; "Thrombolysis", included to capture studies on this essential therapeutic approach in stroke management; "Mechanical Thrombectomy", integrated to ensure the inclusion of research on this innovative procedure; and "Stroke Rehabilitation", incorporated to gather studies on rehabilitation practices and patient outcomes. This strategic selection of terms aimed to balance specificity with comprehensiveness, enabling the identification of studies that were directly aligned with the central themes of our review, as well as those offering valuable peripheral insights.

Selection criteria

Rigorous selection criteria were established to ensure the inclusion of studies that met the highest standards of relevance and quality.

Inclusion criteria encompassed studies directly relevant to advancements in acute ischemic stroke management, including clinical trials, systematic reviews, and meta-analyses. Clinical trials offered insights into the latest intervention strategies, while systematic reviews and meta-analyses provided a comprehensive synthesis of existing evidence.

Exclusion criteria focused on the temporal scope and thematic relevance. Studies outside the 2013-2023 timeframe and those not aligning with the core theme of acute ischemic stroke advancements were excluded to maintain the review's contemporary relevance and coherence.

This methodological rigor ensured that the studies integrated into our review met the stringent criteria of relevance, quality, and timeliness, thereby offering readers a well-synthesized and current perspective on acute ischemic stroke management.

Developments in diagnosis and early management

Advanced Imaging Techniques

Advancements in imaging technologies over the past decade have significantly improved the early and precise diagnosis of acute ischemic stroke. Both MRI and CT, long-established diagnostic tools, have undergone substantial enhancements in resolution, speed, and functionality, greatly augmenting their utility in stroke management [12-16].

MRI has seen notable improvements in specialized imaging modalities, including high-resolution diffusion-weighted imaging (DWI), which facilitates the early and accurate detection of ischemic strokes by identifying restricted water diffusion in acute infarction areas. Perfusion-weighted imaging (PWI) now provides critical insights into cerebral blood flow, aiding in the identification of salvageable brain tissue (ischemic penumbra) and guiding therapeutic decisions. Susceptibility-weighted imaging (SWI) has advanced the detection of microvascular abnormalities and hemorrhagic transformations, essential for assessing treatment eligibility. Innovations like ultrafast MRI protocols have significantly reduced scan times, making MRI more accessible for emergency settings, while MR angiography (MRA) offers a detailed visualization of vascular occlusions, supporting mechanical thrombectomy planning. Additionally, AI integration has enabled the automated detection of ischemic lesions and enhanced real-time decision-making, further refining MRI's role in acute stroke care [12-14].

Similarly, CT imaging has advanced with improvements in resolution, speed, and functional capabilities. High-resolution CT angiography (CTA) now allows for the rapid identification of large vessel occlusions, crucial for determining eligibility for mechanical thrombectomy. CT perfusion (CTP) imaging provides detailed assessments of cerebral blood flow, cerebral blood volume, and time-to-peak perfusion, enabling the precise identification of the ischemic core and salvageable penumbra, even beyond conventional treatment windows. The adoption of whole-brain CTP techniques offers dynamic, volumetric imaging with unprecedented spatial and temporal resolution, enhancing diagnostic accuracy and supporting extended treatment opportunities. Additionally, ultra-low-dose CT protocols and iterative reconstruction algorithms have reduced radiation exposure while maintaining image quality, making CT imaging safer and more efficient for repeated use. AI integration in CT analysis has further enhanced the speed and accuracy of ischemic lesion detection and patient triage in hyperacute scenarios [12,13,15].

These advancements collectively demonstrate transformative progress in imaging technologies over the past decade, enabling timely interventions, expanding therapeutic opportunities, and ultimately improving patient outcomes in acute ischemic stroke management. By leveraging AI-driven workflows, dynamic imaging techniques, and rapid diagnostic capabilities, modern imaging tools have redefined the standards of stroke care, ensuring more effective and personalized management strategies.

Biomarkers: bridging diagnosis and prognosis

The pursuit of reliable biomarkers in ischemic stroke has evolved in tandem with advancements in imaging technologies. Brain natriuretic peptide (BNP) and glial fibrillary acidic protein (GFAP) have risen as prominent candidates in this field, offering the potential to revolutionize both the early diagnosis and prognostication of ischemic stroke [16-19].

BNP's journey from a marker primarily known for cardiovascular regulation to a significant indicator in neurology underscores its versatility. Its elevated levels post-acute ischemic events highlight a nuanced interplay between cardiac and cerebral health. BNP has shown moderate sensitivity (ranging from 65% to 85%) and specificity (from 60% to 80%) in identifying cardioembolic strokes compared to other subtypes. Elevated BNP levels have been correlated with atrial fibrillation, which is a common cause of cardioembolic strokes, underscoring its role in distinguishing this subtype from other stroke mechanisms. This dual connection between cardiac and cerebral health makes BNP a valuable tool for stratifying stroke etiology, particularly in patients where atrial fibrillation may be undiagnosed. However, BNP is not without limitations. Its levels can be influenced by non-neurological factors such as heart failure, renal dysfunction, and pulmonary hypertension, reducing its specificity for ischemic stroke. Additionally, the overlap in BNP levels among different stroke subtypes and other comorbid conditions may complicate its interpretation in clinical settings. Despite these challenges, BNP remains a promising biomarker when used in conjunction with other diagnostic tools, providing a more comprehensive approach to identifying cardioembolic strokes [20].

GFAP, an intrinsic component of the neural framework, rises to prominence following central nervous system damage, including ischemic strokes. Its presence in bodily fluids such as serum and cerebrospinal fluid sparks hope for its utility in early stroke diagnosis. GFAP has demonstrated high specificity (up to 85-95%) for distinguishing ischemic strokes from hemorrhagic strokes due to its release following astrocyte damage, which is more pronounced in hemorrhagic strokes. Its sensitivity, however, varies depending on the timing of sampling and the severity of the stroke, with reported values ranging from 60% to 80% in the early phases of ischemic events. The temporal dynamics of GFAP release make it particularly useful for early differentiation between stroke subtypes, which is critical for guiding appropriate therapeutic interventions. Despite its promise, GFAP has limitations. Its levels can be influenced by other central nervous system pathologies, such as traumatic brain injuries, infections, or neurodegenerative diseases, which may reduce its specificity in certain clinical scenarios. Additionally, the variability in GFAP levels across individuals and stroke severities highlights the need for standardized cut-off values to improve its diagnostic reliability. Furthermore, GFAP is less effective as a standalone biomarker for ischemic stroke detection and is best utilized in combination with other biomarkers or imaging modalities to enhance diagnostic accuracy [21,22].

Pre-hospital care: revolutionizing early stroke response

The pivotal role of rapid intervention in stroke management has led to transformative changes in pre-hospital care, particularly for acute ischemic stroke [23,24]. The advent of MSUs marks a significant leap forward in this domain. These units, equipped with state-of-the-art diagnostic tools, including CT scanners and telemedicine capabilities, represent more than just ambulances; they are mobile medical units capable of initiating stroke diagnosis and management in the field [25-27].

These units epitomize the principle of "time is brain" by offering rapid, on-site diagnosis and communication with specialized stroke centers. This approach significantly reduces the delay in treatment initiation, which is crucial for effective stroke management [28-30]. The impact of MSUs stretches beyond immediate care; they represent a paradigm shift in how stroke care is delivered, highlighting the importance of integrating advanced medical technology with swift, decentralized medical response.

Advancements in treatment: redefining stroke therapy

Thrombolytic Therapy: Enhancing Efficacy and Accessibility

Thrombolytic therapy, particularly alteplase, continues to be the linchpin of acute ischemic stroke treatment [31]. Recent research has delved deep into optimizing thrombolytic protocols, revealing a landscape rich in innovation and potential [32,33]. This research is not confined to tweaking existing protocols; it involves a comprehensive reassessment of how thrombolytic therapy is administered, including dosing, timing, and patient selection. The aim is to broaden the treatment window, thus including a wider range of patients who can benefit from this therapy, thereby mitigating the risk of long-term neurological deficits [32].

The exploration of novel thrombolytic agents is reshaping the future of stroke treatment. These new agents promise not just improvements in efficacy but also reductions in associated risks, offering hope for safer, more effective stroke interventions. Recent advancements in thrombolytic therapy have led to the development of novel agents that aim to improve efficacy while minimizing associated risks such as bleeding complications. Tenecteplase (TNK-tPA), a genetically modified variant of alteplase, has garnered significant attention due to its longer half-life, allowing for single-bolus administration, and its greater fibrin specificity, which reduces systemic bleeding risk. Clinical trials, including the Tenecteplase Versus Alteplase Before Endovascular Therapy for Ischemic Stroke (EXTEND-IA TNK) and the Norwegian Tenecteplase Stroke Trial (NOR-TEST), have shown promising outcomes, with tenecteplase demonstrating comparable or superior efficacy to alteplase in achieving reperfusion and improving clinical outcomes. Additionally, desmoteplase, derived from bat saliva, has been studied for its high fibrin specificity and low neurotoxicity, making it a potential candidate for extending the therapeutic window for thrombolysis. Although earlier trials like the Desmoteplase in Acute Ischemic Stroke (DIAS) and the Dose Escalation of Desmoteplase for Acute Ischemic Stroke (DEDAS) yielded mixed results, ongoing research is exploring optimal dosing and patient selection to maximize its utility. Emerging agents such as pamiteplase and other thrombolytics designed to target resistant clots or work synergistically with mechanical thrombectomy are also under investigation. These agents aim to broaden the applicability of thrombolysis to patients who were previously ineligible due to time constraints or contraindications [34,35].

Mechanical thrombectomy: the new frontier in stroke care

Mechanical thrombectomy has revolutionized the management of acute ischemic stroke, transforming it into a cornerstone of modern stroke care. Since its FDA approval in 2004, significant advancements have been made, particularly over the past decade, enhancing the precision, efficacy, and applicability of this procedure. The introduction and widespread adoption of stent retrievers, such as Solitaire (Medtronic, Dublin, Ireland) and Trevo (Stryker, Kalamazoo, MI, USA), marked a major breakthrough, offering higher recanalization rates, fewer complications, and faster clot retrieval compared to earlier devices like Merci (Inova, Falls Church, VA, USA). These innovations have significantly improved outcomes for patients with severe cerebral occlusions, opening new avenues of hope for those previously deemed ineligible for treatment [36-38].

Clinical trials conducted since 2013, including the Multicenter Randomized Clinical Trial of Endovascular Treatment of Acute Ischemic Stroke in the Netherlands (MR CLEAN), Evaluation Study of Congestive Heart Failure and Pulmonary Artery Catheterization Effectiveness (ESCAPE), Endovascular Revascularization With Solitaire Device vs Best Medical Therapy in Anterior Circulation Stroke Within 8 h (REVASCAT), and Diffusion-Weighted Imaging or CTP Assessment With Clinical Mismatch (DAWN), have demonstrated the efficacy of thrombectomy in extending the treatment window to up to 24 hours for select patients with salvageable brain tissue. This has redefined patient selection criteria, allowing clinicians to leverage advanced imaging techniques, such as CTP and MRI-based penumbra assessments, to better identify candidates who may benefit from the procedure. The integration of AI into imaging analysis has further enhanced procedural planning, enabling faster and more accurate decision-making [36-38].

Technological advancements have also brought aspiration devices and combined techniques (stent retrievers with aspiration catheters) into clinical practice, improving clot retrieval efficiency. The use of balloon guide catheters has optimized flow control, reducing the risk of distal embolization and increasing the success rates of first-pass recanalization. Efforts to refine devices for smaller, distal vessel occlusions are expanding the applicability of thrombectomy to previously untreatable cases [37,38].

The implementation of the "drip-and-ship" model exemplifies the expanded reach of thrombectomy services. By facilitating rapid initial treatment at primary care centers and subsequent transfer to comprehensive stroke centers, this model has effectively bridged the gap between localized healthcare settings and advanced stroke interventions, ensuring timely and equitable access to care for a broader patient population [37,38].

These advancements collectively underscore the transformative progress in mechanical thrombectomy, making it a dynamic and evolving frontier in stroke care. With its ability to provide life-saving intervention and improve long-term outcomes, thrombectomy continues to redefine the landscape of acute ischemic stroke management.

Combination therapies: crafting a synergistic approach

Combination therapies in stroke treatment represent the convergence of thrombolytic and endovascular methods, offering synergistic benefits in acute ischemic stroke management. Approaches such as intravenous thrombolysis with alteplase or tenecteplase followed by mechanical thrombectomy have shown improved reperfusion rates and clinical outcomes, particularly for large vessel occlusions. Bridging therapy, where thrombolytics are administered prior to thrombectomy in a "drip-and-ship" model, ensures timely intervention. Intra-arterial thrombolysis combined with thrombectomy provides localized clot dissolution, reducing systemic bleeding risks. Emerging trends, including dual thrombolytic therapy and the use of neuroprotective agents, aim to optimize clot resolution and protect brain tissue during reperfusion. These advancements exemplify a comprehensive and tailored approach to stroke care [36-38].

The decision-making process in employing combination therapies is increasingly nuanced, informed by a robust evidence base and a deep understanding of individual patient profiles. This personalized approach moves away from a one-size-fits-all methodology, considering factors such as the patient's condition, the type of occlusion, and the timing of intervention.

In conclusion, the current trajectory of acute ischemic stroke treatment is marked by relentless innovation and a commitment to improving patient outcomes. The advancements in thrombolytic therapy and mechanical thrombectomy and the introduction of combination therapies signify a new era in stroke care. These developments are not just expanding the range of therapeutic options but are also reshaping the entire landscape of acute stroke management. As these therapies evolve, they continue to offer new hope and possibilities for patients afflicted.

Post-stroke care and rehabilitation

Rehabilitation Techniques: Tailoring Recovery Pathways

In the realm of post-stroke care, a significant transformation has taken place in rehabilitation techniques, pivoting from a one-size-fits-all approach to a more personalized and patient-centric model. Rehabilitation has evolved to embody a customized journey, aligning with the unique needs and recovery goals of each patient.

Intensive physical therapy has become central to this transformation. Far from a generic set of exercises, it is now a bespoke path tailored to individual patient profiles, targeting specific deficits and fostering functional recovery. The focus extends beyond mere physical recovery to encompass a holistic restoration of dignity and independence [39].

Occupational therapy has similarly broadened its scope. Moving past basic daily living activities, it now encompasses vocational and leisure aspects, helping patients reintegrate into their roles in family, work, and community. Occupational therapists leverage their skills to craft personalized interventions, empowering patients to overcome the limitations imposed by stroke and redefine their identities [40].

Speech therapy has evolved to address not just speech difficulties but also broader cognitive and communicative challenges. Tailored to each patient's unique neurological profile, it aims to enhance understanding, expression, and overall communication, recognizing the diverse impacts of stroke on language and cognition [41].

In essence, modern rehabilitation techniques form a symphony of recovery, each aspect fine-tuned to resonate with the individual's journey towards rehabilitation.

Telemedicine: bridging the distance in stroke care

The rise of telemedicine has marked a new epoch in post-stroke care, particularly beneficial for patients in remote or underserved regions. It has effectively bridged the geographical divide, bringing specialized care to the patient's doorstep. Virtual consultations offer a continuous link between clinicians and patients, allowing for real-time monitoring, therapy adjustments, and timely interventions.

Remote monitoring technologies, including wearable devices and sensors, serve as vigilant guardians of health, continuously tracking vital signs and alerting healthcare providers to potential issues. This ongoing data stream equips clinicians with the necessary tools for proactive care.

Tele-rehabilitation programs stand out in the telemedicine landscape, democratizing access to rehabilitation services. These programs offer patients the flexibility to engage in therapy from their homes, overcoming barriers of distance and mobility, thus making rehabilitation more accessible and inclusive.

Patient-centered care models: a holistic approach

The shift from traditional post-stroke care models to patient-centered care represents a transformative change in stroke management. Traditional models often followed a clinician-driven, standardized approach focused primarily on physical recovery and acute health management, with limited attention to individual patient needs or preferences. In contrast, patient-centered care adopts a holistic strategy that integrates the patient's values, preferences, and overall well-being into the care plan. This approach prioritizes not only medical outcomes but also the overall quality of life for the patient.

A key element of patient-centered care is shared decision-making, which fosters a collaborative relationship between clinicians and patients. This process empowers patients as active participants in their care journey, ensuring that their voices are integral to treatment decisions. By aligning care with individual needs and preferences, this model enhances patient satisfaction and engagement, ultimately contributing to better health outcomes.

Patient-centered care also addresses the broader aspects of recovery, engaging multidisciplinary teams to support physical, cognitive, emotional, and social rehabilitation. Innovations such as telemedicine and personalized rehabilitation programs further enhance the adaptability and inclusivity of this approach, allowing care to be tailored to the unique recovery goals of each individual.

Additionally, these models recognize the essential role of caregivers in the recovery process. By providing caregivers with the necessary support and resources, they acknowledge and strengthen their contribution to the patient's rehabilitation journey. This evolution underscores the importance of addressing the comprehensive spectrum of patient and caregiver needs to improve outcomes and overall quality of life after a stroke.

Challenges and future directions

Despite significant advancements in stroke management, access to advanced treatments continues to be uneven, highlighting persistent healthcare disparities. These inequities are often rooted in broader societal and structural challenges, including lack of health insurance, inadequate access to specialized care, and shortages of healthcare services in rural areas. Socioeconomic disparities further exacerbate these issues, as individuals from lower-income groups may struggle to afford necessary treatments or transportation to healthcare facilities. Additionally, systemic barriers such as limited healthcare infrastructure, uneven distribution of medical resources, and a shortage of trained professionals in underserved regions hinder timely diagnosis, treatment, and rehabilitation. Collectively, these challenges disproportionately impact marginalized populations, leading to poorer health outcomes and emphasizing the need for targeted interventions to bridge these gaps. Bridging these gaps necessitates a holistic strategy, including policy reforms and targeted interventions aimed at reducing structural barriers, thereby promoting equitable access to stroke care for all individuals.

The integration of AI and machine learning is transforming stroke management by introducing advanced tools for diagnosis, risk prediction, and early intervention. By analyzing large-scale data, these technologies uncover complex patterns and subtle indicators, significantly improving diagnostic accuracy and stroke risk prediction. Their potential to revolutionize care makes them indispensable in modern stroke management. However, successful implementation requires addressing critical challenges, including establishing robust ethical guidelines to address data privacy, algorithmic bias, and transparency. Equally important is comprehensive clinician training to ensure the accurate interpretation of AI-driven insights and seamless integration into clinical practice. By overcoming these barriers, AI can be deployed effectively and equitably, ultimately improving patient outcomes and advancing stroke care standards.

Addressing key research gaps, particularly in understanding minor strokes (such as transient ischemic events) and the long-term effects of emerging therapies, remains a critical priority. Novel neuroprotective agents, including hypothermia-inducing drugs and antioxidants, hold promise for minimizing ischemic damage and promoting neuroregeneration. Similarly, innovative rehabilitation techniques, such as brain-computer interface systems and virtual reality-based therapies, show potential to enhance motor and cognitive recovery in stroke patients. These advancements, combined with patient-centered care and tailored rehabilitation programs, not only improve physical and cognitive outcomes but also address psychosocial factors, aiding reintegration into daily life. Furthermore, advanced diagnostic tools and effective management of risk factors reduce stroke recurrence rates and improve overall quality of life. Together, these approaches offer a pathway to significantly improving long-term outcomes for stroke patients and advancing care strategies.

## Conclusions

Telemedicine has significantly improved access to post-stroke rehabilitation, particularly in underserved regions. Evidence suggests that tele-rehabilitation programs, such as those utilizing wearable devices for remote physical therapy or virtual platforms for speech and cognitive exercises, enable continuous monitoring and personalized care. Studies have demonstrated that stroke patients participating in tele-rehabilitation achieve recovery outcomes comparable to or better than those receiving traditional in-person therapy, especially in areas with limited access to specialized rehabilitation centers. Additionally, telemedicine reduces barriers such as travel time and costs, offering the flexibility of at-home therapy and ensuring that more patients can access the care they need to enhance recovery outcomes. However, challenges such as disparities in technology adoption and data privacy concerns must be addressed for broader implementation. Multidisciplinary approaches remain vital to post-stroke rehabilitation, combining the expertise of neurologists, therapists, and caregivers to address the diverse needs of patients. These approaches ensure that treatment plans are comprehensive and tailored to each patient's unique challenges and recovery goals. Personalized care models, which emphasize shared decision-making and active caregiver involvement, foster greater patient engagement and adherence to rehabilitation protocols. This collaborative strategy not only improves functional recovery and reintegration into daily life but also empowers patients and caregivers, enhancing their overall satisfaction and quality of life.

Advanced technologies like AI are transforming stroke management through enhanced diagnosis and risk assessment. AI-driven tools for diagnosis, risk assessment, and early intervention rely on large datasets to identify patterns with unprecedented accuracy. Despite these advances, ethical and regulatory concerns remain, including algorithm transparency, biases in datasets, and compliance with global health data regulations like the General Data Protection Regulation (GDPR). Addressing these issues is essential for ensuring the equitable and effective application of these technologies in clinical settings.

Recent clinical trials for neuroprotective drugs represent a promising frontier in stroke management. Several types of neuroprotective agents are under investigation, including hypothermia-inducing drugs, antioxidants such as edaravone, and N-methyl-D-aspartate (NMDA) receptor antagonists. Additionally, agents targeting excitotoxicity, inflammation, and oxidative stress, such as glutamate release inhibitors and anti-inflammatory cytokines, are being explored. These drugs aim to minimize neuronal damage, promote neuroregeneration, and enhance functional recovery. These trials focus on agents aimed at minimizing ischemic damage and promoting neuroregeneration, with early-phase results showing potential efficacy in reducing infarct size and enhancing recovery. To maximize their impact, it is crucial to expand these trials to larger and more diverse populations, enabling the development of comprehensive clinical guidelines and protocols.

The expansion of stroke care networks, including MSUs and regional centers, has significantly reduced treatment delays and improved access in underserved areas. Studies indicate that MSUs can reduce the time to treatment (door-to-needle time) by an average of 15-30 minutes compared to standard emergency services, which is critical given the "time is brain" principle in stroke care. This reduction in time has been associated with better patient outcomes, including higher rates of functional independence at 90 days and decreased mortality. Similarly, the establishment of regional stroke centers has facilitated faster triage and specialized care, leading to improved access to advanced interventions like thrombolysis and mechanical thrombectomy. However, scaling these initiatives requires significant investment in infrastructure and workforce training. Potential strategies to address these barriers include leveraging public-private partnerships to fund infrastructure development, implementing telemedicine and mobile health technologies to extend reach, and establishing training programs or certification courses to upskill healthcare workers. Additionally, policy reforms and financial incentives could encourage the allocation of resources to underserved regions, ensuring equitable access to advanced stroke care.
